# Magnitude of post-operative mortality and associated factors among patients who underwent surgery in Wolaita Sodo teaching and referral hospital, SNNPR region, Ethiopia

**DOI:** 10.4314/ahs.v21i4.42

**Published:** 2021-12

**Authors:** Tiwabwork Tekalign, Habtamu Balta, Lolemo Kelbiso

**Affiliations:** School of Nursing, College of Health Science and Medicine, Wolaita Sodo University, Wolaita Sodo, Ethiopia

**Keywords:** Magnitude, mortality, surgery, post-operative, Wolaita

## Abstract

**Background:**

Each year 4.2 million people around the world die within 30 days of surgery and postoperative deaths account for 7.7 % of all deaths. So this study aimed to asses' magnitude of postoperative mortality and associated factors among patients who underwent surgery in Wolaita Sodo University Teaching referral Hospital.

**Method:**

Retrospective cross sectional design was carried out from April 15–30 2019. Card review was done on 384 participants by using Systematic sampling technique. Entered to Epi Data; exported to SPSS for analysis. Variables with p-value < 0.25 in bivariate analysis were entered to multivariate logistic regression. Statistical significance is determined at p-value < 0.05.

**Results:**

The magnitude of postoperative mortality was 5.7%. Using surgical check list (AOR= 0.18; 95% CI 0.05 to 0.61), having comorbid condition (AOR= 4.45; 95% CI 1.39 to 14.19), and don't having blood transfusion (AOR= 0.07; 95% CI 0.02 to 0.22) and general anesthesia (AOR= 4.37; 95% CI 1.17 to 16.30) are factors of post-operative mortality.

**Conclusion:**

The magnitude of postoperative mortality was high. Surgical check list, comorbidity, blood transfusion and general anesthesia are factors. The hospital should encourage using of surgical check list and work on comorbid patients to decrease the mortality.

## Introduction

Operative mortality refers to any death, regardless of cause, occurring within 30 days after surgery, in or out of the hospital, and after 30 days during the same hospitalisation subsequent to the operation^1^.

Surgery is an essential component of health care systems and currently the overall burden of disease that may be cured, palliated or treated with surgical intervention is large and rapidly growing^2^.

Globally, 313 million surgical procedures are performed each year. In low- and middle-income countries (LMICs), 60% of surgical operations are performed for patients who need emergency surgery, But 143 million additional surgical procedures are needed each year to save lives and prevent disability^3^, ^4^.

Surgical procedure is not risk-free and it always comes with a risk of death due to the procedure or the anaesthesia that is given during the procedure and also due to postoperative complications with considering patient condition at the time of surgery^5^, ^6^.

Surgical care affects the lives of millions of people. Studies indicate that complications following surgery result in disability or a prolonged hospital stay in 3–25% of hospitalised patients, depending on the complexity of surgery and the hospital setting, In fact of this 28–32% of the global burden of disease can be attributed to surgically treatable conditions^7^, ^8^.

Although each year, 4.2 million people around the world die within 30 days of surgery, and postoperative deaths account for 7.7 % of all deaths globally, half of these occur in LMICs^9^. A study conducted in St Paul's Hospital Millennium Medical College, Addis Ababa, showed that surgical mortality rate was 3.41%^10^. Besides in Ethiopia, both a shortage of surgeons and surgical care make the problem worse^11^, ^12^.

Currently, surgical mortality is considered as one indicator of quality of care, so this study aims to assess magnitude of postoperative mortality and associated factors among patients who underwent surgery in Wolaita sodo university teaching and referral hospital

## Methods and material

### Study setting, design and population

Wolaita Sodo University Teaching Hospital (WSUTH) is located in South Nations Nationalities and People Region States (SNNPRS), Ethiopia. The hospital was established in 1928 and serves 2 million people. Retrospective cross sectional study design was carried out. All and selected medical records of patients who underwent surgery in year of 2008–2010 E.C in WSUTRH were taken as a source population and study population respectively.

### Variables of the study

In this study magnitude of mortality is considered as dependent variable whereas Socio-demographic Characteristics (Age, Sex, Occupation, Marital status, place of residence) and general health profile (types of surgery, types of anesthesia, length of stay, surgical check list, blood transfusion, and comorbidity) were independent variables.

### Eligibility criteria

All medical record of patient who underwent surgery during the study period were included in the study while incomplete medical record were excluded from the study

### Sample size determination

The sample size was determined by using a single population proportion formula. The following assumptions were applied: p, prevalence of 50%(since there is no locally conducted study), d is the expected margin of error (5%), Z, the standard score corresponding to a 95% confidence interval and α, the risk of rejecting the null hypothesis (0.05). Accordingly the required sample size became 384.

### Sampling technique

A total of 6623 patients underwent surgery in year of 2008–2010 E.C in WSUTRH. From them using systematic sampling technique in every seventeenth interval, a total of 384 patient cards were identified and traced using registration number.

### Data collection procedure/instruments

Data were collected by using pretested checklist from April 1–30 2019. All the variables of interest were assessed accordingly and the checklist was prepared in English. Those who have diploma in nursing were participated in the data collection process. Training was given to the data collectors.

### Data Analysis

Epi data version 3.5.1 used for data entry and SPSS version 21 was used for analysis. Descriptive statistics were computed to determine frequencies and summarize statistics. Variables having P-value <0.25 in bivariate analysis were selected as a candidate for multivariate logistic regression. Finally P value < 0.05, at 95% confidence interval was declared as statistically significant.

### Data quality control

Orientation and appropriate supervision were done to data collectors by supervision made on daily basis by the principal investigator. And completeness and consistency were checked every day during data collection. Pre- test was done on 5% of the total sample size in the same hospital on patient records before the study period. Appropriate modifications were made after analyzing the pre-test result before the actual data collection.

## Results

### Socio-Demographic Characteristics

A total of 384 patient medical records were enrolled in this study. Out of the total respondents, a majority 182(47.4%) were in age between^21^^–40^. More than half 200 (52.1%) and 216, 56.3% of participants were of female and living in rural respectively. Two hundred and seventy four (71.4%) were married and 238 (62.0%) were unemployed (see Table 1).

**Table 1:** socio-demographic characteristics of study subjects undergone surgery in WSUTRH in last from 2008–2010 E.C (n=384)

Variables	Category	Frequency (%)
Age of participant	0–20 years	73(19.0)
	21–40 years	182(47.4)
	41–60 years	84(21.9)
	>60 years	45(11.7)
Residence	Rural	216(56.3)
	Urban	168(43.8)
Sex	Male	184(47.9)
	Female	200(52.1)
Marital status of patients	Single	75(19.5)
	Married	274(71.4)
	Others ^1^	35(9.1)
Occupational status	Employed	146(38.0)
	Unemployed	238(62.0)

Others ^1^- divorced, widowed

### General Health Profile of Study Participants

Respectively, 354(92.2%) and 340 (88.5%) of the participants didn't have a postoperative surgical checklist and comorbid conditions. Three fourth 288(75.0%) and 61 (15.9%) of participants stayed in the hospital for less than a week and have blood transfusions history, respectively. Two hundred and sixty two (68.2%) u1`nderwent emergency surgery, and more than half >1(52.3%) received regional anaesthesia (see Table 2).

**Table 2:** General health profile of study participants who come for surgery in WSUTRH in last from 2008–2010 E.C (n=384)

Variables	Category	Frequency (%)
Surgical check list	Yes	30(7.8)
	No	354(92.2)
Comorbidity	Yes	44(11.5)
	No	340(88.5)
Length of stay	<=1week	288(75.0)
	>1week	96(25.0)
Blood transfusion	Yes	61 (15.9)
	No	323(84.1)
Type of surgery	Elective	122(31.8)
	Emergency	262(68.2)
Type of anesthesia	Regional	201(52.3)
	General	183(47.7)

### Magnitude of Postoperative Mortality

According to this study magnitude of postoperative mortality was 5.7%.

### Associated Factors of Postoperative Mortality

There were 9 variables in binary logistic regression that had a p-value of less than 0.25 and became a candidate for multiple logistic regressions. In multiple logistic regressions, four variables were significantly associated with postoperative mortality, with P value <0.05 (see Table 3).

**Table 3:** bivariate and multivariate analysis of socio demographic and General health profile of study participants who underwent surgery in WSUTRH from 2008–2010 E.C (n=384)

Variable	Category	Postoperative mortality		COR, 95%CI	P- value (<0.25)	AOR,95%CI
		Yes	No			
Occupation	Employed	4(2.7%)	142(97.3%)	0.34(0.11,1.03)	0.152	2.58(0.70, 9.46)
	Unemployed	18(7.6%)	220(92.4%)	1		1
Marital status	Single	6(8.0%)	69(92.0%)	1		1
	Married	11(4.0%)	263(96.0%)	0.48(0.17,1.34)	0.070	0.81(0.20, 3.18)
	Others	5(14.3%)	30(85.7%)	1.91(0.54,6.77)	0.936	0.92(0.13, 6.29)
Residence	Rural	17 (7.9%)	199(92.1%)	1		1
	Urban	5(3.0%)	163(97.0%)	2.78(1.00, 7.71)	0.067	2.99(0.92, 9.68)
Surgical check list	Yes	7 (23.3%)	23(76.7 %)	6.87(2.55, 18.54)	**0.006**	0.18(0.05,0.61)
	No	15(4.2%)	339(95.8%)	1		1
Comorbidity	Yes	7(15.9%)	37(79.4%)	2.44(0.09, 0.63)	**0.012**	4.45(1.39, 14.19)
	No	15(4.4%)	325(95.6%)	1		1
Length of stay	<=1week	11(3.8%)	277(96.2%)	1		1
	>1week	11(11.5%)	85(88.5%)	0.30 (0.12,0.73)	0.523	0.68(0.21, 2.17)
Blood transfusion	Yes	15(24.6%)	46(75.4%)	1		1
	No	7(2.2%)	316(97.8%)	14.7(5.69, 38.02)	**0.000**	0.07(0.02, 0.22)
Type of surgery	Elective	12(9.8%)	110(90.2%)	1		1
	Emergency	10(3.8%)	252(96.2%)	2.74(1.15,6.55)	0.171	2.09 (0.72, 6.04)
Type of anesthesia	General	19(10.4%)	164(89.6%)	7.64(2.22, 26.29)	**0.028**	4.37(1.17,16.30)
	Regional	3(1.5%)	198(98.5%)	1		1

Those having surgical check list were 82% less likely to die postoperatively (AOR= 0.18; 95% CI 0.05 to 0.61) than those who didn't have a postoperative surgical checklist. Those having comorbid conditions were four times to die post-operatively (AOR= 4.45; 95% CI 1.39 to 14.19) than their counterparts. Those didn't have blood transfusion were 93% less likely to die post-operatively (AOR= 0.07; 95% CI 0.02 to 0.22) than those who have blood transfusions. Those patients had given general anaesthesia were four times to die postoperatively (AOR= 4.37; 95% CI 1.17 to 16.30) than those who had given regional anaesthesia.

## Discussion

The overall magnitude of postoperative mortality in this study was 5.7%, higher than the studies carried out at Zewditu memorial Hospital, Addis Ababa, (2.82%), Tikur Anbesa specialized hospital, Addis Ababa (4.5%), in Japan (2%), and Netherlands (1.85%)^13^–^15^. This might be due to a shortage of safe and timely surgical care, as well as the fact that the studied hospital serves a huge catchment area.

However, it was significantly lower than the rate shown by studies conducted in Nigeria (8.3%) and United states (7.0%),^17^, ^18^. This might be due to variations in study duration, socio-demographic characteristics of participants and urbanisation levels of the study area.

Using surgical check list has relationship with postoperative mortality. Those having surgical check list were 82% less likely to die postoperatively (AOR= 0.18; 95% CI 0.05 to 0.61) than those who didn't have a postoperative surgical checklist.

This finding is consistent with studies done in different parts of the world; this may be an indicator of countries that use a World Health Organization (WHO) surgical safety checklist, as recommended^19^–^23^.

Previous history of comorbidities was significantly associated with postoperative mortality. In this study those patients having comorbid conditions were four times to die post-operatively (AOR= 4.45; 95% CI 1.39 to 14.19) than their counterparts. Different studies also indicated that patients having one or more comorbidities were associated with a high risk of mortality from causes other than surgery and related treatment; even it worsens the effect on vital organs^24^–^26^.

Blood transfusion has an impact on postoperative mortality. This study indicated that, those didn't have history of blood transfusion were 93% less likely to die post-operatively (AOR= 0.07; 95% CI 0.02 to 0.22) than those who have blood transfusions. This finding is consistent with studies conducted in the different parts of the world2^4^, ^25^, and 27–30.

Possibly, blood transfusion during an operation is an indicator of a complication. However, this is inconsistent with studies conducted in Veterans Affairs medical Centre^31^. A possible explanation may be that blood transfusions are considered critical for patients undergoing major surgery on one or more of their internal organs because of postoperative blood loss was significant for those patients^32^

In this study those patients had given general anaesthesia were four times to die postoperatively (AOR= 4.37; 95% CI 1.17 to 16.30) than those who had given regional anaesthesia. This is consistent with study conducted America^33^ in which using general anaesthesia is associated with increased postoperative mortality. Surgical procedures which need general anaesthesia by nature are major surgery; association of postoperative death may be related with surgery type in addition to anaesthesia type used.

## Conclusion

According to this study postoperative mortality was high compared to other studies. Using surgical check list, having comorbidity, having blood transfusion and using general anesthesia are predictors.. The hospital should encourage using of surgical check list and work on comorbid patients to decrease the mortality.

## Limitation of the study

The main limitation of this studies its retrospective nature, due to the design we are unable to asses some variables like income, and the type of comorbid condition a patient has.

## Abbreviations/acronyms

• AOR- Adjusted Odds Ration

• CI- Confidence Interval

• COD- Crude Odds Ratio

• SPSS- Statical Package for Social Science

• WSUTH- Wolaita Sodo University Teaching Hospital

## Declaration

### Availability of supporting data

All data supporting the study is available with the reasonable request.
